# Giant Conductivity Modulation and Chemical Neuromodulation via Proton‐Electron Coupling in a Hydrogen‐Bonded Coordination Polymer

**DOI:** 10.1002/advs.75420

**Published:** 2026-04-24

**Authors:** Kwangmin Park, Jumin Park, Huiyeong Ju, Nasim Arafat, Byoung Gwan Lee, Joohee Oh, Eejin Jang, Hyunseob Lim, Seok Min Yoon, Dae‐Woon Lim, Intek Song

**Affiliations:** ^1^ Department of Chemical and Biological Engineering Gyeongkuk National University Andong Republic of Korea; ^2^ Research Center for Materials Analysis Korea Basic Science Institute Daejeon Republic of Korea; ^3^ Department of Chemistry Yonsei University Wonju Republic of Korea; ^4^ Department of Chemistry Gwangju Institute of Science and Technology Gwangju Republic of Korea; ^5^ Center for Quantum Conversion Research Institute for Basic Science Gwangju Republic of Korea; ^6^ Department of Chemistry Gyeongsang National University Jinju Republic of Korea; ^7^ GIST InnoCORE AI‐Nano Convergence Institute for Early Detection of Neurodegenerative Diseases Gwangju Institute of Science and Technology Gwangju Republic of Korea

**Keywords:** de‐doping, hydrogen‐bonded coordination polymer, mixed protonic‐electronic conductors, neuromodulation, neuromorphic devices, proton‐electron coupling

## Abstract

Mixed protonic‐electronic conductors (MPECs) have been developed to maximize static conductivity for electrochemical applications, but emerging applications that leverage proton‐electron coupling (PEC) require dynamic conductivity control. To achieve this, we propose a “de‐doping” strategy in a hydrogen‐bonded coordination polymer {[Co(DMF)_2_(H_2_O)_2_(bipy)](NO_3_)_2_·2(DMF)}_n_ (bipy = 4,4’‐bipyridine, DMF = *N,N*‐dimethylformamide) named Co‐BAND. By isostructural substitution of Ni(II) (*d* ^8^) in the established Ni‐BAND with Co(II) (*d* ^7^), we designed Co‐BAND to suppress the intrinsic conductivity while preserving proton transport and PEC. As a result, Co‐BAND exhibits a giant conductivity modulation (1.15 × 10^6^) in response to humidity changes and implements complex brain‐like learning rules. We also demonstrate chemical control of synaptic plasticity via solvent vapor exposure. This biomimetic neuromodulation tunes transport and learning rules based on vapor polarity, proticity, and steric effects. This work establishes conductivity modulation as an important design metric for MPECs and highlights their potential as designable platforms for stimuli‐responsive applications.

## Graphical abstract

A “de‐doping” strategy positions mixed protonic–electronic conductors (MPECs) as adaptive neuromorphic platforms with dynamically tunable transport. Co‐BAND achieves giant conductivity modulation (>10^6^) and chemically tunable synaptic plasticity. Analogous to biological neuromodulation, solvent vapors dynamically reprogram the device's learning rules based on molecular polarity and proticity, enabling environment‐adaptiveness.

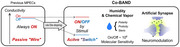

## 1 Introduction

Dynamic modulation of conductivity is essential for high‐level functionality in both artificial and biological systems. In modern electronics, the significance of semiconductors stems not from high absolute conductance as seen in metals, but from their ability to switch electron flow [1, 2]. Similarly, biological and bio‐inspired systems utilize controlled proton transport in response to external stimuli for signal transduction, sensing, and energy conversion [3]. Integrating these two transport modes in a single system bridges the gap between these regimes [4, 5, 6, 7]. Realizing this integration requires materials capable of supporting coupled protonic and electronic responses. Consequently, recent studies on single‐material mixed protonic‐electronic conductors (MPECs) have identified them as compelling candidates [5, 8, 9, 10, 11].

MPECs are distinguished from simple mixtures of two conductors by the presence of proton‐electron coupling (PEC) in transport, a macroscopic analogue of proton‐coupled electron transfer (PCET) [5]. This coupled transport is inherent to general mixed conductors because any movement of ions creates a local charge imbalance that must be neutralized by electronic carriers [8, 12]. In MPECs, specifically, deprotonation generates a negatively charged framework, which the system compensates for via n‐type doping [5, 8]. Such doping by coupling accounts for the high conductivity of MIECs (e.g., PEDOT:PSS) and MPECs (e.g., Ni‐BAND) [8, 13]. The unique characteristics of protons further enrich MPECs’ functionality. For example, the rapid Grotthuss hopping of protons along hydrogen bond networks enables a strong structure‐property correlation, contrasting with slow and stochastic motion typical of other ions [5, 9, 11]. Moreover, the inherent sensitivity of proton transport to moisture makes humidity an efficient handle to control transport [10]. Designable molecular platforms in which external stimuli control electronic and protonic transport are already well established in areas such as spin‐crossover compounds and responsive coordination frameworks [14, 15, 16]. These precedents suggest that MPECs may also be developed as platforms in which external stimuli dynamically control both protonic and electronic transport [8].

**FIGURE 1:**
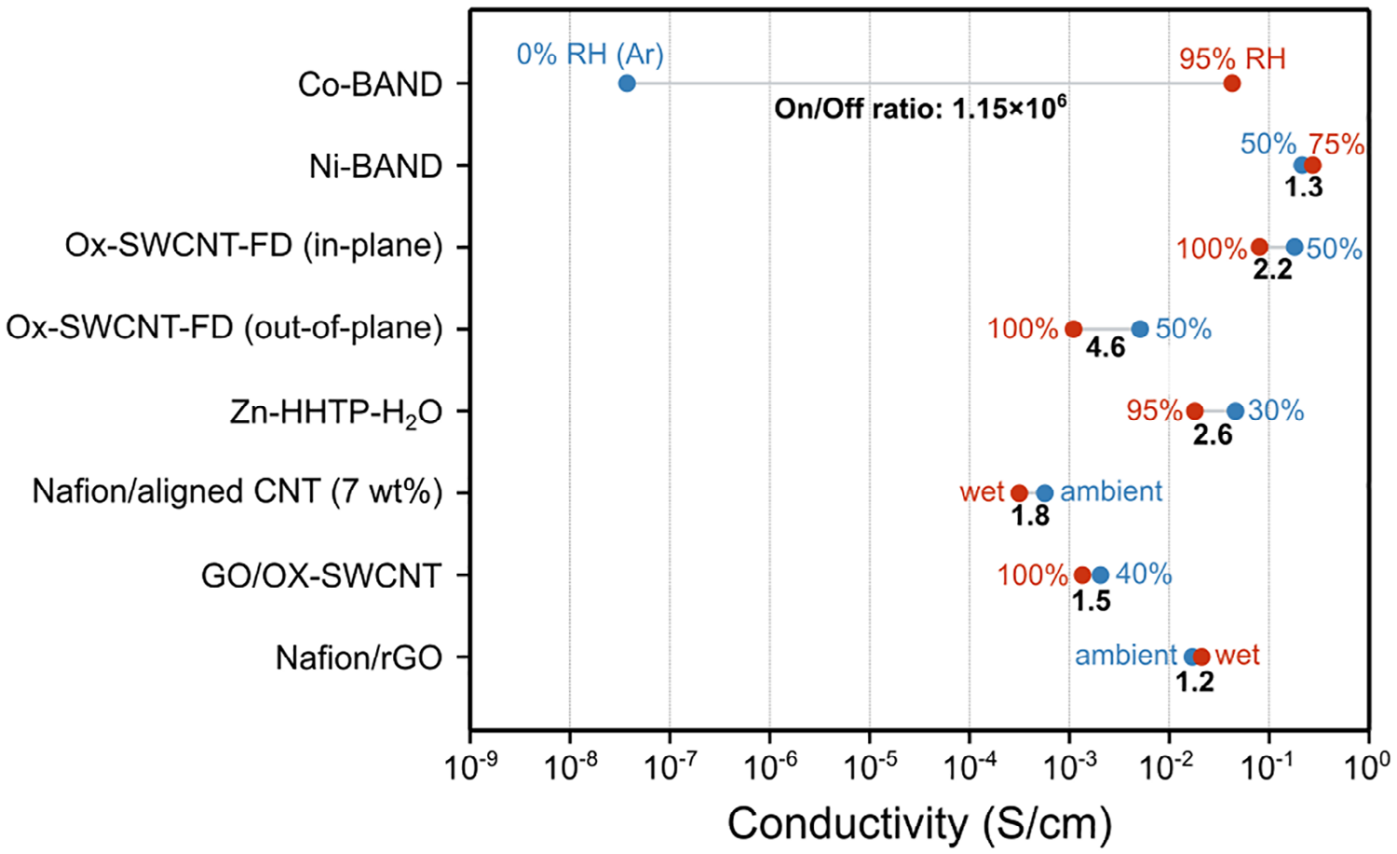
Electrical conductivity modulation of mixed protonic‐electronic conductors under varying humidity levels. The blue numbers denote relative humidity (RH) at the blue dot (least conductive, “off” state), and the red numbers that of the red dot (most conductive, “on” state). The bold face numbers below the gray horizontal lines denote the on/off ratio. *Source*: Ni‐BAND [5], Ox‐SWCNT‐FD [18], Zn‐HHTP‐H_2_O [11], Nafion/aligned CNT (7 wt.%) [19], GO/Ox‐SWCNT [20], Nafion/rGO (5%) [21]. Note that Ni‐BAND shows the minimal conductivity at 50% RH.

However, the prevailing design paradigm for MPECs has prioritized the maximization of static conductivity, limiting dynamic tunability [5, 8, 9]. This trade‐off is exemplified by our previously reported hydrogen‐bonded coordination polymer, Ni‐BAND [5]. Ni‐BAND behaves like a metal, maintaining a nearly constant “always on” conductivity state. For example, it only shows a 30% increase via humidity control and a ∼2‐fold change in synaptic devices. Although stimulus‐responsive switching is broadly established in other classes of molecular materials, analogous design strategies for achieving large and reversible conductivity modulation in single‐material MPECs remain comparatively underdeveloped (Figure 1) [5, 11, 17, 18, 19, 20, 21]. As a result, many reported MPECs have functioned primarily as high‐conductivity transport media rather than as materials deliberately engineered for large, reversible switching responses. Advancing MPEC functionality, therefore, requires materials with low baseline conductivity and strong PEC for dynamic modulation.

To achieve this, we propose a “de‐doping” strategy to suppress baseline conductivity while preserving the structural motifs essential for PEC. Given that PEC induces an n‐doping effect in MPECs, reducing the initial electron density of the system can maximize the impact of n‐doping by PEC, thereby achieving a high on/off ratio [22]. As a model system to validate this concept, we substituted the Ni(II) centers (*d* ^8^ electronic configuration) of Ni‐BAND with Co(II) (*d* ^7^). The similar electronegativity (1.84 for Co vs. 1.88 for Ni) and ionic radii (89 pm for six‐coordinated Co^2+^ vs. 83 pm for Ni^2+^) preserve the structural motifs for proton transport and PEC [23].

**FIGURE 2:**
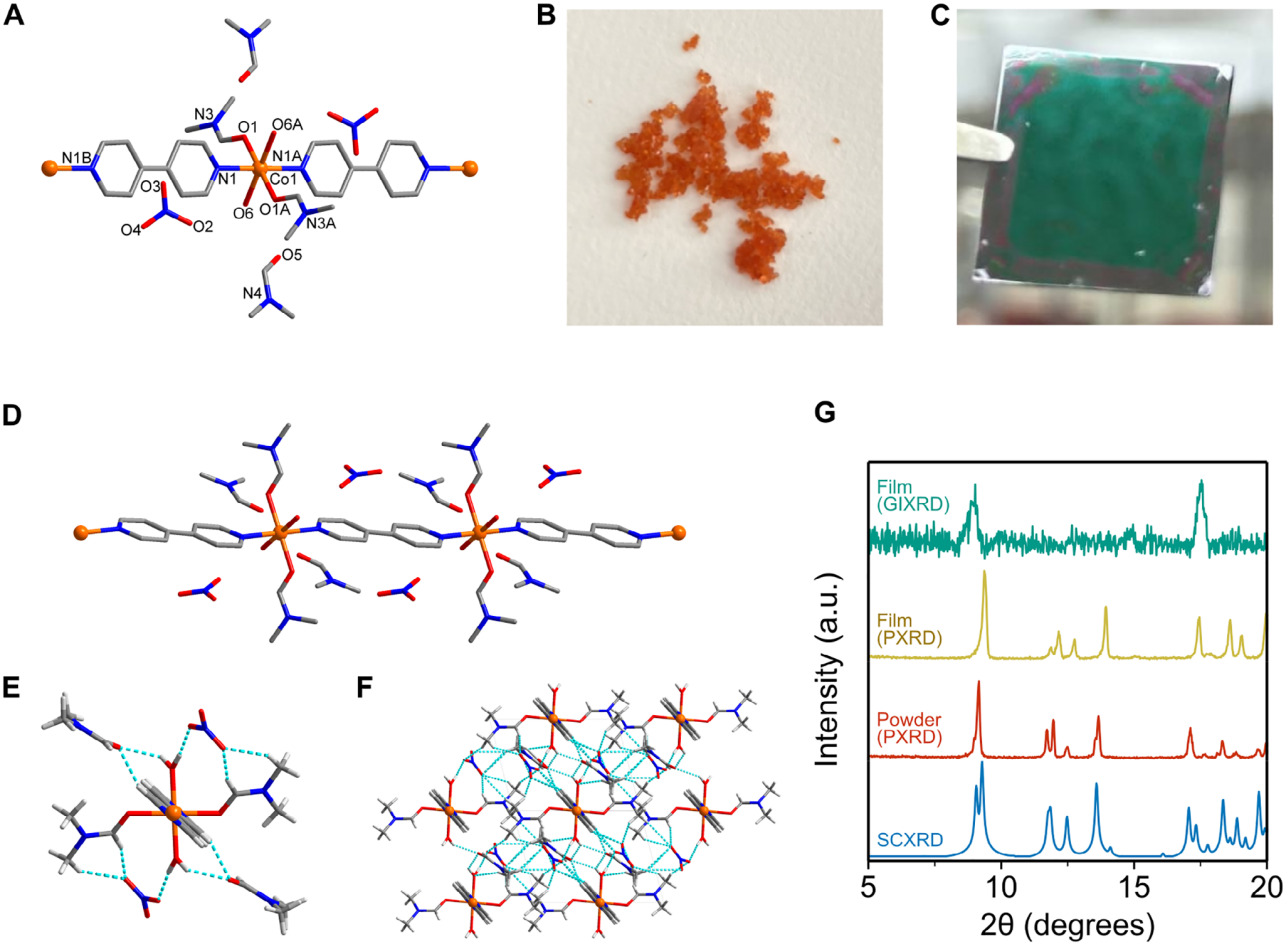
Synthesis and structural characterization of Co‐BAND (A) The unit cell structure of Co‐BAND. (B) and (C): Photographs of Co‐BAND crystals (B) and spin‐coated thin film on a SiO_2_(300 nm)/Si substrate (C). (D) Crystallographic structure showing bipy‐Co‐bipy 1D chains. (E) and (F): Illustration of hydrogen bond networks (cyan dashed lines) within a unit cell (E) and intercellular connection (F). (G) X‐ray diffraction patterns of Co‐BAND: calculated pattern from single‐crystal X‐ray diffraction (SCXRD) data (blue), experimental powder pattern (red), thin‐film powder diffraction pattern (yellow), and thin‐film grazing‐incidence diffraction pattern (green).

Herein, we report Co‐BAND {[Co(DMF)_2_(H_2_O)_2_(bipy)](NO_3_)_2_·2(DMF)}_n_ (bipy = 4,4’‐bipyridine, DMF = *N,N*‐dimethylformamide), a hydrogen‐bonded coordination polymer that exemplifies this design principle (Figure 2A). Co‐BAND demonstrates ultralow dry‐state electrical conductivity (∼3.72 × 10^−8^ S cm^−1^) yet achieves high proton (∼1.80 × 10^−2^ S cm^−1^) and electron (∼4.26 × 10^−2^ S cm^−1^) conductivity under humid conditions (25°C and 95% RH). This modulation spans six orders of magnitude, which, to the best of our knowledge, is among the largest reported for single‐phase, non‐nanostructured materials without post‐synthetic modification. Based on the coupled transport, Co‐BAND bridges the electronic and bio‐inspired regimes by emulating complex brain‐like learning rules, including long‐term potentiation/depression (LTP/LTD) and paired‐pulse facilitation (PPF), where protons act as “neurotransmitters”. Beyond water, exposure to various chemical vapors further modulates transport and neuromorphic properties depending on their polarity, proticity, and steric effects. Each vapor molecule effectively functions as a synthetic “neuromodulators” that regulate the transport of the protonic neurotransmitters. This work highlights conductivity modulation as an important design metric for MPECs, positioning them as promising platforms for environmentally responsive and intelligent systems.

## 2 Results

### 2.1 Synthesis and Structural Characterization

The precursor for Co‐BAND was first synthesized by slow evaporation of an ethanolic solution of Co(NO_3_)_2_∙6H_2_O and bipy [24]. This pink‐colored precursor was re‐dispersed in DMF, and the dispersion slowly precipitated orange‐colored crystallites of Co‐BAND over 3 days (Figure 2B). Spin coating the as‐prepared dispersion onto solid substrates like SiO_2_/Si, quartz, or ITO‐coated glasses yielded Co‐BAND thin films with a thickness of approximately 450 nm (Figure 2C; Figure S1).

Single‐crystal X‐ray diffraction (SCXRD) analysis revealed that Co‐BAND crystallizes in the triclinic space group (*P*‐1) with the following unit cell parameters: *a* = 8.439(7) Å, *b* = 10.287(8) Å, *c* = 10.407(8) Å, *α* = 93.67(2)°, *β* = 111.49(2)°, *γ* = 105.46(3)°, and *V* = 797.1(11) Å^3^ (CCDC Reference: 2454283). The Co(II) center adopts an octahedral coordination geometry, defined by two H_2_O ligands, two DMF ligands, and two bridging bipy ligands at the *trans* positions (Figure 2A). The Co‐N1(bipy) bonds are slightly longer at 2.158(3) Å, the Co‐O1(DMF) bonds measure 2.111(3) Å, and the Co‐O6(H_2_O) bonds are shorter at 2.082(3) Å. The bridging bipy has nearly parallel pyridinyl rings and links adjacent Co(II) centers into 1D chains of bipy‐Co‐bipy (Figure 2D).

These 1D chains assemble into a 3D network via an extensive hydrogen‐bonding network. Applying Steiner's criteria for hydrogen bonds in solids (H···A bond length <3 Å and X‐H···A angle >110° for X‐H···A bonds), there are 12 unique contacts per unit cell, including relatively weak C─H···O bonds (Table S1) [25]. For example, the coordinated H_2_O ligands act as hydrogen‐bond donors to the adjacent NO_3_^−^ anion (O6···O2 distance = 2.727 Å) and non‐coordinating DMF molecules (O6···O5 distance = 2.840 Å) (Figure 2E). The hydrogen bonds further interconnect adjacent bipy‐Co‐bipy chains, establishing extensive hydrogen bond networks between the chains (Figure 2F)

The experimental X‐ray diffraction patterns of both the bulk powder and the thin film are consistent with the simulated pattern from SCXRD data, confirming sample uniformity (Figure 2G). Also, grazing incidence X‐ray diffraction (GIXRD) of the thin film shows matching diffraction patterns. Based on the thermogravimetric analysis (TGA), Co‐BAND shows no weight loss up to 80°C, above which sequential losses of water and DMF occur at 100°C and 150°C, respectively (Figure S2). Also, Co‐BAND displays negligible uptake of N_2_, confirming its non‐porous structure (Figure S3).

### 2.2 Proton‐Electron Coupled Transport and its Modulation

**FIGURE 3:**
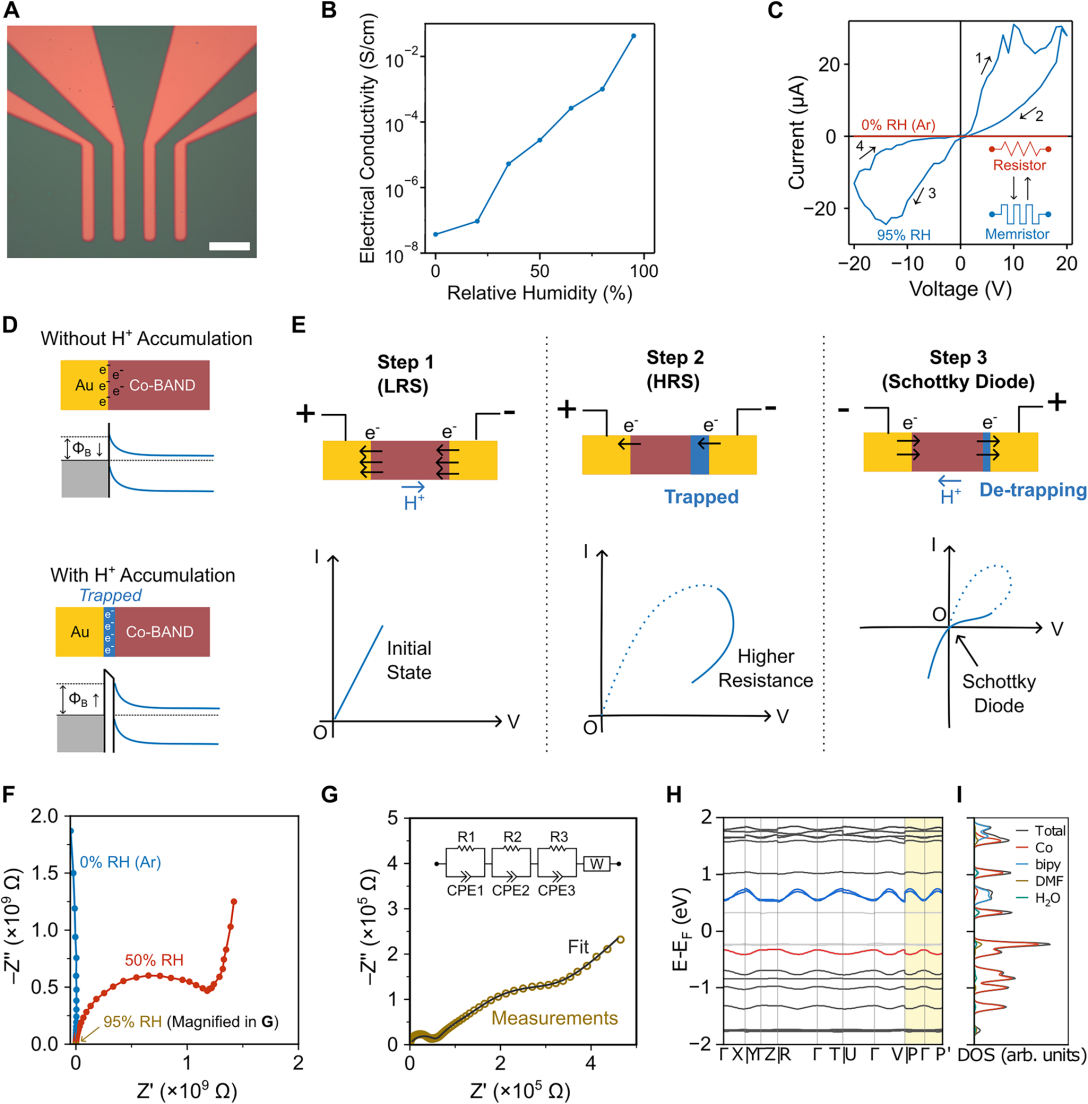
Proton‐electron coupled transport and memristive properties of Co‐BAND (A) A photograph of an electrical device. The scale bar denotes 200 µm. (B) Electrical conductivity measured by four‐point probe measurements. (C) *I‐V* sweep characteristics of two‐point probe devices under dry Ar conditions (red) and at 95% RH (blue). The digitized arrows show the direction of the *I‐V* sweep. (D) Variation of the Schottky barrier at the Co–BAND/Au interface as a function of interfacial proton accumulation. (E) Schematic I–V characteristics and the corresponding spatial distributions of protons and electrons for each step are shown in (C). (F) Electrochemical impedance spectra measured under dry Ar conditions (blue), 50% RH (red), and 95% RH (yellow). (G) Magnified view of the impedance spectrum under 95% RH (yellow curve in (F)), with corresponding fit and equivalent circuits (the inset). Fitting parameters: R1 = 7.21 × 10^4^ Ω; CPE1 = 8.56 × 10^−8^ Ω^−1^s^α^ (α = 7.52 × 10^−1^); R2 = 1.06 × 10^5^ Ω; CPE2 = 1.02 × 10^−7^ Ω^−1^s^α^ (α = 9.87 × 10^−1^); R3 = 5.67 × 10^4^ Ω; CPE3 = 1.74 × 10^−9^ Ω^−1^s^α^ (α = 7.01 × 10^−1^); W = 5.66 × 10^5^ Ω s^−1/2^. (H) Calculated band structure of Co‐BAND using density functional theory. The reciprocal lattice is shown in Figure S10. (I) Calculated partial density of states (PDOS) of the band structures corresponding to (H).

To investigate the charge carrier transport properties, we fabricated thin film devices using chemically inert Au electrodes (Figure 3A). To ensure accurate electrical characterization, we employed a four‐point probe technique to eliminate contact resistance. This approach is critical for mixed conductors, as ionic accumulation at the interface can introduce significant contact resistance, causing two‐point probe DC measurements to deviate from the intrinsic material properties [5, 26, 27]. Additionally, we recorded current only at the steady state to exclude potential artifacts arising from ionic flow (Figure S4). Under dry Ar conditions, the device shows a very low direct current (DC) conductivity of 3.72 × 10^−8^ S cm^−1^, showing the insulating nature of pristine Co‐BAND (Figure 3B). Additionally, the current‐voltage (*I‐V*) sweep did not show any pinched hysteresis (red curve in Figure 3C).

However, introducing humidity exponentially increased DC conductivity with relative humidity (RH), rising by 1.15 × 10^6^ times to 4.26 × 10^−2^ S cm^−1^ at 95% RH (Figure 3B). This magnitude of humidity‐driven conductivity modulation is among the highest in single‐phase, non‐nanostructured materials without post‐synthesis modification (Table S2) [28, 29, 30, 31, 32]. The *I‐V* sweep also exhibits a large, pinched hysteresis at this condition, indicating resistive switching behavior consistent with memristor functionality (blue curve in Figure 3C) [33].

The conductivity increase is accompanied by water vapor adsorption at 298 K, exhibiting a type IV‐like profile, mirroring the adsorption behavior of non‐porous Nafion and Ni‐BAND (Figure S5) [5, 34]. Ex situ PXRD pattern of Co‐BAND powder exposed to 95% RH matches well with the PXRD patterns of the as‐received specimens and SCXRD references, indicating reversible sorption without disrupting long‐range crystallinity (Figure S6). Since additional water molecules cannot coordinate to Co(II), the water uptake can be attributed to framework swelling driven by hydrophilic groups (H_2_O, DMF, and NO_3_^−^) and hydrogen bond networks along the 1D bipy‐Co‐bipy chains, supplemented by adsorption at surfaces and grain boundaries [3, 35, 36]. At higher RH, more water molecules are cooperatively adsorbed through hydrogen bond networks throughout the interchain space. This behavior mirrors that of isostructural Ni‐BAND, non‐porous hydrophilic polymers, and hydrogen‐bonded organic framework composites, where substantial hydration occurs through transient hydrogen‐bonded water layers and reversible local swelling that preserves the structure over varying RH [3, 37, 38, 39].

At 95% RH, the Arrhenius plot of the electrical conductivity exhibits a nonlinear correlation between ln(*σ*) and 1/*T*, which is a stark contrast to the linear correlation of semiconductors (Figure S7). Differential thermal analysis (DTA) study confirms the absence of phase changes within this temperature range, indicating that the observed nonlinearity is not a result of phase transition or other structural changes (Figure S2). This deviation suggests that under high humidity, the electron transport mechanisms originate from a process fundamentally different from that of typical semiconductors.

To understand the correlation between the electrical conductivity and humidity, we performed electrochemical impedance spectroscopy (EIS) (Figure 3F). Under dry conditions, the Nyquist plot shows a vertical line like a capacitor, aligning with the insulating property of Co‐BAND [40]. As RH increases, the spectrum transforms to show a large high‐frequency semicircle and a low‐frequency Warburg tail, indicating the onset of mixed transport of protons and electrons [27]. By 95% RH, where the adsorption isotherm suggests multilayer water formation, the spectrum features a small high‐frequency semicircle and a pronounced Warburg tail (Figure 3G). The real impedance intercept at the low‐frequency limit (corresponding to two‐point‐probe DC resistance) was not fully resolved due to the limited frequency range (1 Hz–1 MHz), which is commonly observed in other MPECs and MIECs [5, 27, 41]. The estimated proton conductivity is 1.80 × 10^−2^ S cm^−1^, and the activation energy derived from the Arrhenius plot is 0.33 eV, supporting the Grotthuss mechanism (Figure S8). Density functional theory (DFT) calculations suggest that these mobile protons can originate from the water ligands in Co‐BAND, which possess a lower proton dissociation energy than bulk water (Table S3).

These observations demonstrate that humidity effectively modulates proton transport in Co‐BAND. At low RH, the proton transport is suppressed, because the hydrogen bonding network between bipy‐Co‐bipy chains contains C─H···O type bonds, which are incapable of transferring protons by the Grotthuss mechanism. As humidity increases, water adsorption occurs through the network, leading to a progressive rise in proton conductivity. At high RH, substantial water adsorption establishes a continuous hydrogen‐bonded pathway, thereby enabling efficient proton transport via the Grotthuss mechanism [3, 10, 35]. Since protons cannot penetrate gold electrodes, the accumulation of protons at the interface results in an electrical double layer (EDL), appearing as a Warburg tail in the impedance spectra (Figure 3G).

The observed giant electrical conductivity modulation via humidity control is driven by proton transport and PEC. Under humid conditions, deprotonation of the framework generates negatively charged sites, which induce n‐type doping to maintain charge neutrality. This “compensation doping” mechanism is well established in MIECs, where the migration of an ion generates the doping by the opposite charge for charge neutrality [5, 8]. A notable example is highly conductive PEDOT:PSS, where heavy p‐doping arises from PSS anion transport [5, 8, 13]. In general, MIECs, such as doped states, typically manifest as mid‐gap states resulting from polaron formation [8, 42]. Consistent with this, UV–vis–NIR spectroscopy of Co‐BAND under ambient conditions reveals additional absorption features between 1000 and 2000 nm (Figure S9). Since the DFT calculations, which correspond to the dry‐state limit, predict an optical gap of ∼1 eV (∼1240 nm), these additional peaks are reasonably attributed to mid‐gap states, similar to polarons in MIECs (Figure 3H; Figure S10). Consequently, the n‐doping by PEC enables conduction in de‐doped Co‐BAND through the dispersed conduction band dominated by bipy (Figure 3I).

This dynamic doping mechanism explains the material's anomalous temperature dependence. Co‐BAND's nonlinear correlation between ln(*σ*) and 1/*T* is typical of MIECs and MPECs (Figure S7) [43]. Since proton transport is thermally activated, higher temperature induces more n‐type doping in the system [8]. This synergistic effect leads to a sharper increase in conductivity compared to standard semiconductors, resulting in an apparent activation energy that exceeds the transport gap. Moreover, mixed conductors often display a nonlinear correlation between the charge carrier mobility and the charge carrier density due to structural disorders [8].

Finally, the proposed transport mechanism accounts for the pinched hysteresis observed in Co‐BAND. This hysteresis appears only under humid conditions, highlighting the essential role of mobile protons [10]. As shown in the *I‐V* curves (blue curve in Figure 3C), the device gradually shifts from a low‐resistance state (LRS) to a high‐resistance state (HRS) under a sustained bias (Steps 1→2 and 3→4), but it returns to the LRS immediately when the bias polarity is reversed (Steps 2→3 and 4→1). This behavior is attributed to proton migration that dynamically modulates an asymmetric Schottky barrier at the Co‐BAND/Au interface (Figure 3D). A similar switching mechanism driven by interfacial traps has been reported in MoS_2_ memristors, where mobile defects lead to charge trapping at the interface [44, 45]. Under a positive bias (Step 1), protons drift toward the interface of the negative electrode, creating a Schottky junction under a “reverse‐bias condition” (Figure 3E). As more protons accumulate (Step 2), the interfacial barrier height increases, which progressively reduces conductance (LRS → HRS) [4]. When the bias polarity is reversed (Step 3), the accumulated protons redistribute away from the interface (de‐trapping), shifting the Schottky diode under a “forward‐bias condition” to display a gradual increase in conductance (HRS → LRS). The same sequence explains the remaining sweep (Step 4) with the opposite polarity.

### 2.3 Synaptic Plasticity and Chemical Neuromodulation

**FIGURE 4:**
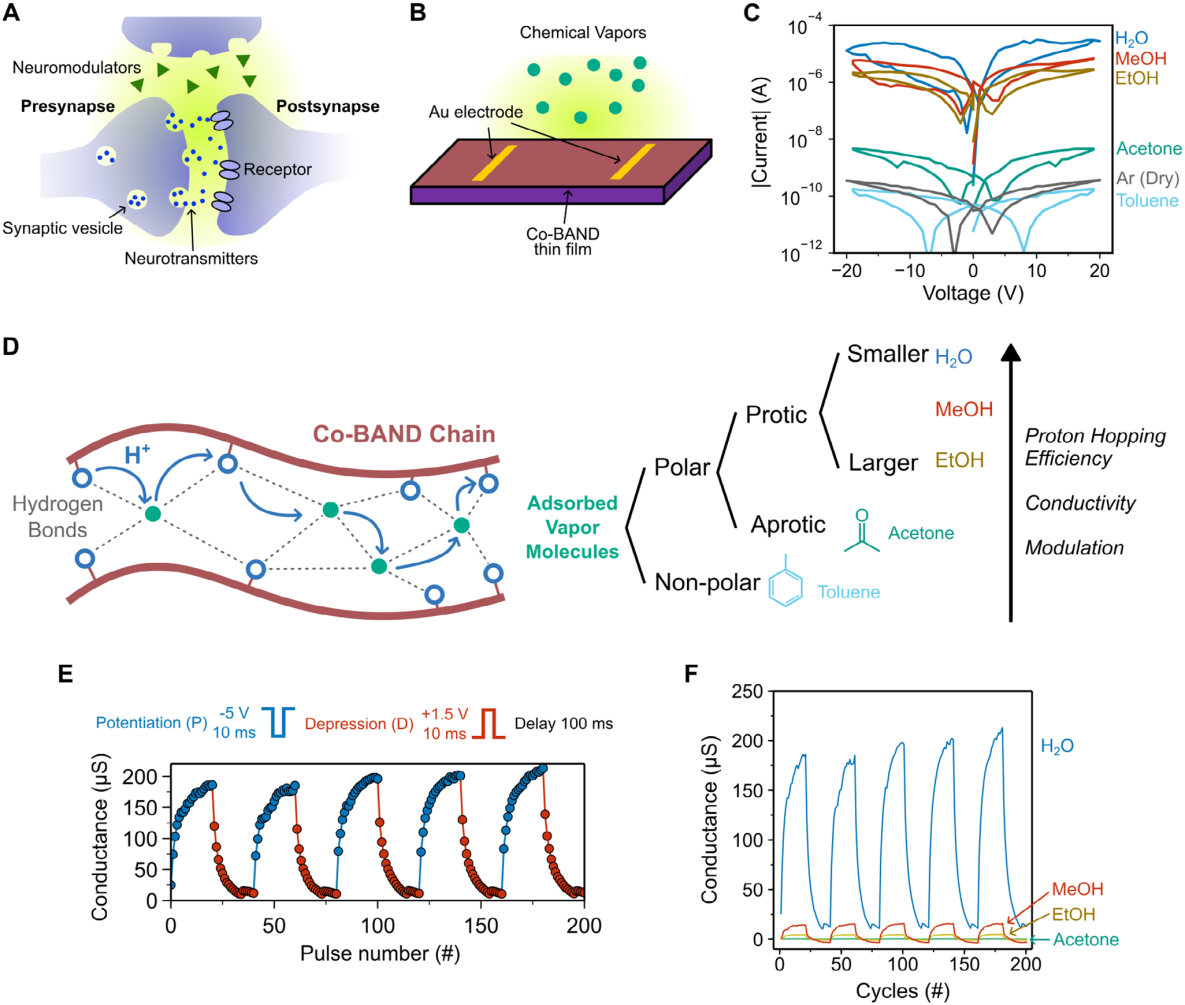
Chemical neuromodulation in Co‐BAND synaptic devices. (A) Schematic illustration of biological neurons and synapses, highlighting the role of neuromodulators. (B) Schematic diagram of Co‐BAND artificial synaptic devices, where chemical vapors function analogously to neuromodulators. (C) *I‐V* sweep characteristics showing pinched hysteresis loops under exposure to different chemical vapors. The specific vapor used is indicated to the right of the curves. The graph corresponding to water vapor (blue) is identical to the blue curve (95% RH) in Figure 3C. (D) Illustration of adsorbed vapor molecules (green) and the established hydrogen bond network for protons (blue), with the molecular characteristics of the vapors and their correlation with bulk transport properties. (E) Long‐term potentiation (LTP) and depression (LTD) behaviors of the Co‐BAND device. The specific pulse conditions are shown above. (F) Modulation of LTP/LTD characteristics in Co‐BAND devices under various chemical vapor exposures, with the specific gas indicated above. The applied pulse conditions and the water graph (blue) are identical to those in (E). Abbreviations: MeOH, methanol; EtOH, ethanol.

The presence of pinched hysteresis is a key feature for artificial synaptic devices, as it emulates biological synapses for neuromorphic computing (Figure 4A,B) [33, 46]. Neuromorphic computing has recently gained much attention because of its potential to realize energy‐efficient artificial intelligence (AI) and brain‐machine interfaces (BMIs) that support coherent interaction between the human brain and machine [47, 48, 49]. Notably, Co‐BAND exhibits memristive properties exclusively under humid conditions, creating a bio‐realistic system that mirrors the aqueous environment of biological organisms. In Co‐BAND synaptic devices, protons effectively serve as “neurotransmitters”. This behavior exemplifies Co‐BAND's capacity to function as active switches for proton‐electron coupled transport, bridging electronic and protonic regimes.

To elucidate this, we applied a series of electrical pulses to simulate the learning rules in biological neurons. When the Co‐BAND device was subjected to a series of set pulses, its conductance gradually increased (potentiation), and it was reduced by the reset pulses (depression). The Co‐BAND device achieved a conductance on/off ratio of ∼40 during these potentiation/depression cycles at 95% RH, showing ∼20‐fold improvement over the Ni‐BAND gel‐based devices (Figure S11) [5]. Furthermore, the device showed long‐term potentiation and depression with a 100 ms delay between set (reset) pulses and read pulses (Figure 4E). Paired‐pulse facilitation (PPF) was also demonstrated, where the identical second pulse induces a stronger response (Figure S12).

While proton transport and PEC in MPECs are commonly associated with water, other chemical vapors also mediate proton transport and PEC in Co‐BAND. This suggests that the modulation of synaptic plasticity by external vapors mimics the role of neuromodulators in biological neurons, offering a chemically configurable neuromorphic platform [50, 51]. To investigate this conductivity modulation, the *I‐V* sweep measurements were conducted upon exposure to various solvent vapors with different proticity, polarity, and steric effects (Figure 4C). Polar solvent vapors (water, methanol, ethanol, and acetone) increased conductivity and produced a pinched hysteresis in the *I‐V* sweeps. By contrast, both the non‐polar solvent vapor (toluene) and dry Ar produced near‐baseline device behavior; the small difference between these two conditions is not considered meaningful, as both signals lie within the sub‐nA region, the measurement limit of our setup. Among the polar solvents, the protic types (water, methanol, and ethanol) resulted in a highly conductive pinched hysteresis, but aprotic acetone showed only a small increase in conductance. Steric effects were also significant among the polar protic solvents. Small water molecules resulted in the highest conductivity, while the bulkiest ethanol showed the lowest.

These results demonstrate that efficient proton transport at the molecular level directly translates into PEC‐driven conductivity enhancement and resistive switching behavior (Figure 4D). The negligible effect of non‐polar vapors confirms this correlation, since they do not interact with the hydrogen bond network. The reduced impact of aprotic polar solvents is attributed to their inability to donate protons, which is essential for the Grotthuss mechanism [52]. Finally, the material's negligible porosity sterically prohibits the adsorption of bulkier molecules through hydrogen bond networks, explaining their smaller increase in conductivity. This clear correlation shows how microscopic molecular properties directly manifest macroscopic transport phenomena.

Based on these observations, we demonstrate that molecular‐level chemical interactions between the vapor and Co‐BAND framework can reprogram the macroscopic learning rules in Co‐BAND. In LTP/LTD, water showed the highest on/off ratio and conductance, followed by methanol, ethanol, and acetone (Figure 4F) [53, 54]. Moreover, each vapor environment requires distinct pulse conditions for optimal operation. For example, methanol vapor subjected to water‐optimized pulse conditions exhibited negative conductance due to a non‐zero‐crossing effect, where proton accumulation and capacitive effect cause the abnormal current flow (red curve in Figure 4F) [53, 54]. In contrast, pulse parameters optimized for ethanol vapor substantially reduced the on/off ratio of water‐exposed devices (Figure S13). These findings indicate that chemical vapors do not simply shift the baseline conductance but fundamentally reconfigure the charge transport properties of the materials. This behavior parallels the role of neuromodulators, including dopamine (DA), in biological synapses as modulators for neurotransmission in potentiation and depression, establishing Co‐BAND as a chemically controllable artificial synapse [50, 51]. This mechanism is also distinguished from previous reports on an inorganic memristor that relies on surface redox reactions, rather than molecule‐level chemical interaction [55, 56]. This makes Co‐BAND unique among memristive systems, as it achieves chemical modulation of synaptic plasticity within a single‐phase crystalline framework through rationally designed proton–electron coupled transport, enabling predictable control via molecular‐level chemical interactions rather than interfacial or surface phenomena.

## 3 Conclusion

In conclusion, we have established a de‐doping design strategy to develop Co‐BAND, whose conductivity is strongly controlled by humidity, electrical pulses, and chemical vapors. Through isostructural substitution of the metal center, we achieved reduced intrinsic dry‐state conductivity while preserving the hydrogen bond network critical for PEC. This approach achieves six orders of magnitude modulation in conductivity in response to humidity and enables the robust implementation of neuromorphic learning rules. Furthermore, the system emulates chemical neuromodulation, where the molecular properties of vapors govern the macroscopic transport and neuromorphic behaviors. By demonstrating that large‐amplitude, reversible transport control can be rationally designed in an MPEC, this work broadens the design framework of proton–electron‐coupled materials beyond static conduction and toward adaptive information‐processing functionalities.

## 4 Experimental Section/Methods

### 4.1 Synthesis and Preparation of Crystals and Thin Films

The precursor for Co‐BAND was initially synthesized by dissolving 5 mmol of Co(NO_3_)_2_·6H_2_O (Sigma–Aldrich, 98%) and 5 mmol of 4,4’‐bipyridine (Sigma–Aldrich,98%) into 6 mL of ethanol (Acros organics, 99.8%). The precursor immediately began to form after mixing, further promoted by sonication. The precipitated precursor was collected for further steps.

For crystal synthesis, the precursor was dispersed into dimethylformamide (Alfa Aesar, 99.9%) to achieve a concentration of 0.15 g mL^−1^. The solution was sealed in a vial and left undisturbed at room temperature for around 72 h to precipitate orange‐colored crystals. The crystals were then filtered through a nylon filter.

For thin film preparation, the dispersion was prepared at 0.15 g mL^−1^, which is identical to that used in the crystal growth. It was directly spin‐coated onto various substrates, including SiO_2_ (300 nm)/Si (Namkang Hi‐tech), quartz (Hi‐Solar), and ITO glass (ITO pattern glass, Taewon Scientific). Spin‐coating was conducted at 800 rpm for 10 s, followed by 2000 rpm for 60 s, resulting in a film of ∼450 nm in thickness. Prior to spin coating, the substrate was treated with air plasma using a plasma cleaner (Alpha Science, PDC‐32G‐2) to enhance the film uniformity.

### 4.2 Crystallographic and Structural Characterizations

Optical microscopy images were collected using a Nikon Eclipse LV150NL microscope. Thermogravimetric analysis (TGA) was performed on a Rigaku TG‐8120 instrument. N_2_ adsorption–desorption isotherms were measured using a Microtrac MRB BELSORP‐MAX surface area and porosity analyzer. UV–vis diffuse reflectance spectra were obtained with a Perkin–Elmer Lambda 950 spectrometer. Water vapor sorption isotherms were recorded at 298 K using the Belsorp‐Max instrument (Microtrac BEL Corp., Japan). The temperature was maintained at 298 K using a refrigerated‐heating bath circulator (RW3‐0525, JEIO TECH).

The crystal structure of the crystallized sample was determined by SCXRD methods at the Korea Basic Science Institute (KBSI, Korea). A single crystal was picked up with paratone oil and mounted on a Bruker D8 Venture PHOTON III M14 diffractometer equipped with a graphite‐monochromated Mo Kα (*λ* = 0.71073 Å) radiation source and a cold stream (−50°C). Data collection and integration were performed using the software package of APEX3 (Bruker) [57]. The absorption correction was performed by a multi‐scan method implemented in SADABS [58]. The structure was solved by direct method using the SHELXS program of the SHELXTL package and refined by full‐matrix least‐squares methods with SHELXL‐2018 [59]. All the non‐hydrogen atoms were refined anisotropically, and the hydrogen atoms were added to their geometrically ideal positions. Powder X‐ray diffraction was performed using a Rigaku Ultima IV instrument equipped with CuKα radiation (2 kW).

### 4.3 Electron and Proton Transport Characterizations

Thin film devices were fabricated by depositing 25 nm thick gold electrodes onto Co‐BAND films using a thermal evaporator (Daedong High Technologies, Model Bridge) and shadow masks to define the device geometry. All electrical transport measurements were conducted using a Nextron MPS‐VAC probe station equipped in a Jeiotech TH3‐KE chamber for precise temperature and humidity control. DC electrical measurements, including two‐ and four‐probe conductivity and neuromorphic pulse sequences, were measured using a Keithley 2636B SourceMeter. To measure electrical conductivity, a four‐point‐probe measurement was applied, where a constant current was applied to the outer electrodes, and the bias difference was measured in the inner electrodes. The voltage and current values were utilized for conductivity calculation only after the conductance reached the steady state. The electrochemical impedance spectroscopy (EIS) data were collected using a Solartron Model 1260A frequency response analyzer. Prior to further analysis, the linearity, causality, and stability of each spectrum were confirmed with the Kramers–Kronig test [40]. The equivalent circuit modelling and fitting were performed by using the impedance.py package [60].

### 4.4 Theoretical Calculations

Spin‐polarized density functional theory (DFT) calculations were performed using the Vienna Ab‐initio Simulation Package (VASP) [61, 62] to optimize the crystal structure and calculate fundamental electronic properties. The cell volume and ionic positions were optimized based on the crystal structure determined by SCXRD methods with fixed cell shape. The exchange‐correlation energy was evaluated using the Perdew–Burke–Ernzerhof (PBE) [63] functional. The core electrons were replaced by projector augmented wave (PAW) [64] pseudopotentials, expanded in a basis set of plane waves up to a cutoff energy of 400 eV. Electronic and ionic relaxations were performed until the total energy and atomic forces converged below thresholds of 10^−6^ eV and 0.02 eV/Å, respectively. A Γ‐centered 7 × 5 × 5 k‐point grid was used for Brillouin zone sampling. The VASPKIT package [65] was used for the post‐processing of the electronic band structure and density of states.

## Funding

This work was supported by the National Research Foundation of Korea (NRF) (RS‐2021‐NR057845, RS‐2023‐00217555, RS‐2024‐00455116, RS‐2026‐25474092, and RS‐2026‐25486849) and the InnoCORE program of the Ministry of Science and ICT(GIST InnoCORE KH0860).

## Conflicts of Interest

The authors declare no conflicts of interest.

## Supporting information




**Supporting File**: advs75420‐sup‐0001‐SuppMat.pdf.

## Data Availability

The data that support the findings of this study are available from the corresponding author upon reasonable request.
