# Supplementary material for: Giant Conductivity Modulation and Chemical Neuromodulation via Proton‐Electron Coupling in a Hydrogen‐Bonded Coordination Polymer
Source: Adv Sci (Weinh). 2026 Apr 24;13(40):e75420. doi: 10.1002/advs.75420 (PMC13335467; doi:10.1002/advs.75420)
Supplement: Supplementary file 1 — Supporting File: advs75420‐sup‐0001‐SuppMat.pdf. [file ADVS-13-e75420-s001.pdf]

Supporting Information

Giant Conductivity Modulation and Chemical Neuromodulation via Proton-Electron Coupling in a Hydrogen-Bonded Coordination Polymer

Kwangmin Park, Jumin Park, Huiyeong Ju, Nasim Arafat, Byoung Gwan Lee, Joohee Oh, Eejin Jang, Hyunseob Lim, * *Seok Min Yoon,* * *Dae-Woon Lim,* * *and Intek Song* *

*Corresponding authors: hslim17@gist.ac.kr, smyoon@gnu.ac.kr, limdaewoon@yonsei.ac.kr, songintek@gknu.ac.kr

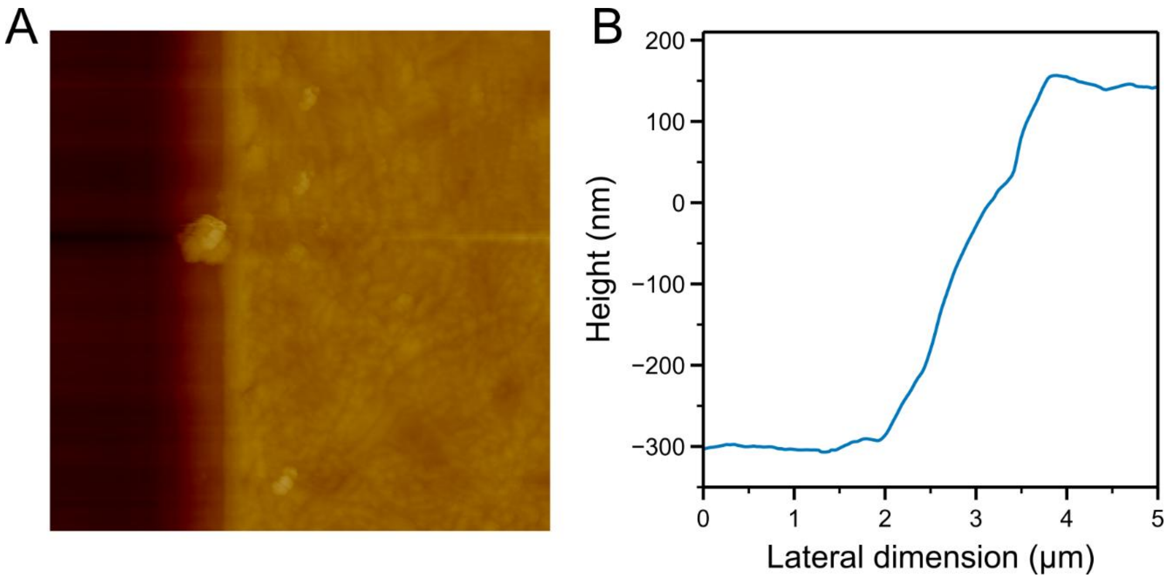

Figure S1. Cross-section profile of spin-coated thin film of Co-BAND. (A) Atomic force microscopy images of the Co-BAND thin film. **(B)** The cross-section profile.

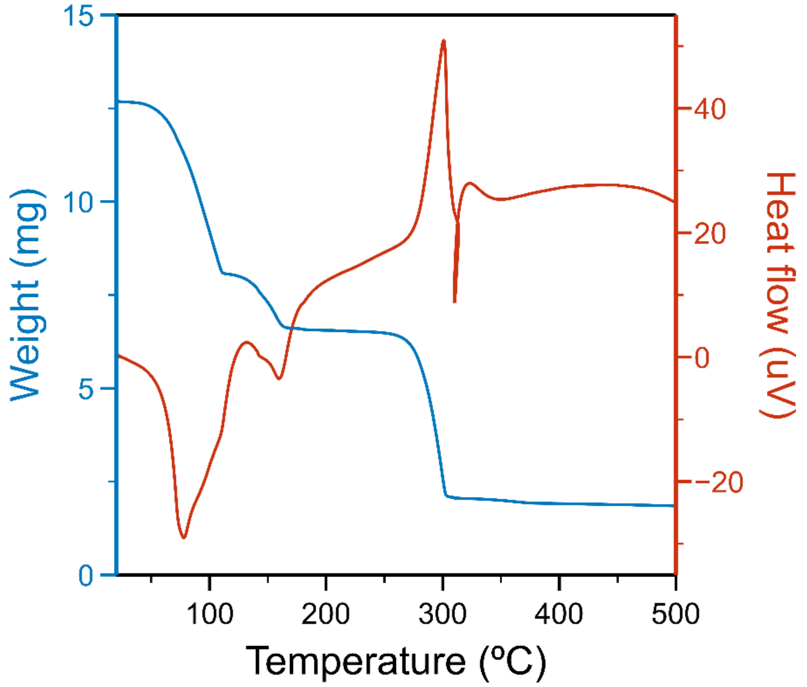

Figure S2. Thermogravimetric analysis data of Co-BAND.

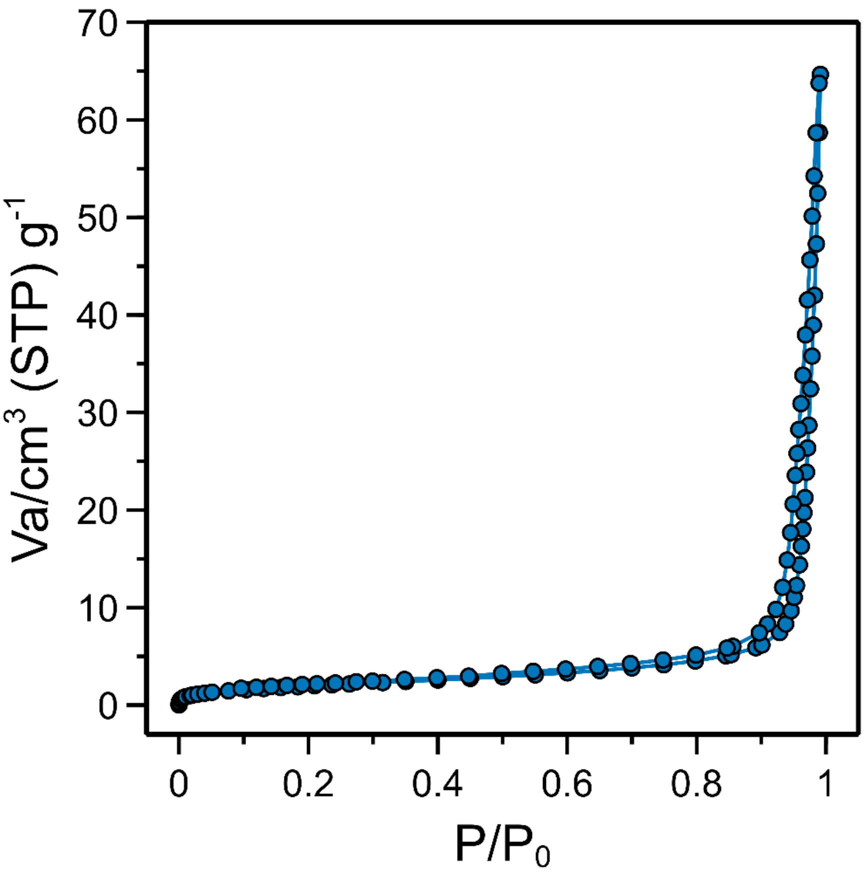

Figure S3. The N_2 vapor adsorption and desorption isotherm.

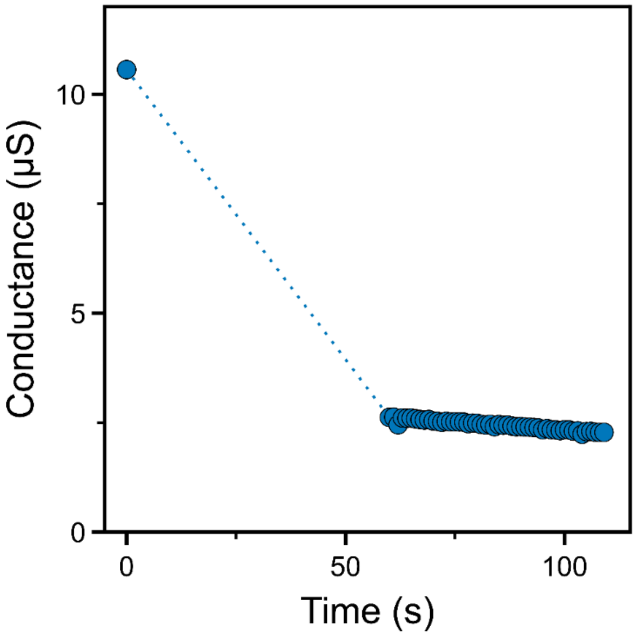

Figure S4. Gradual decay of DC conductance due to ionic flow. A constant current bias was applied throughout the measurement. Data acquisition (voltage reading) was suspended during the initial 0-60 s interval to minimize system perturbation.

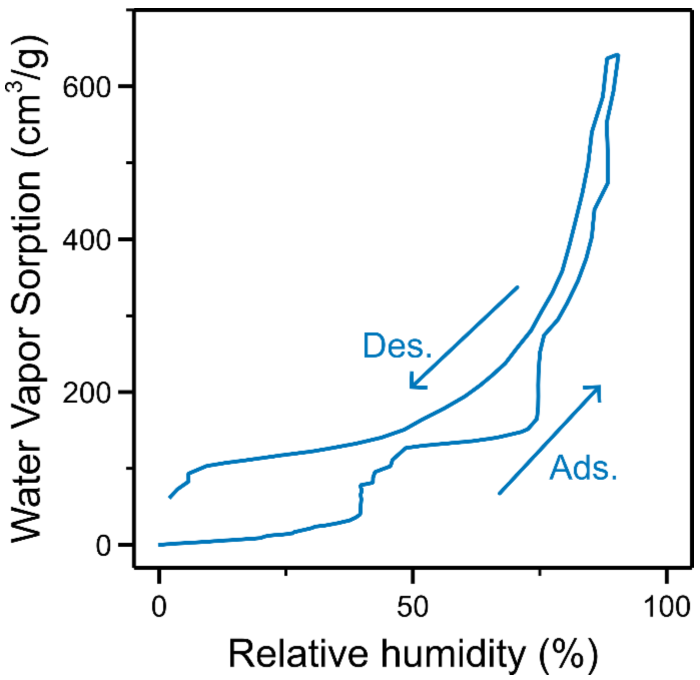

Figure S5. Water vapor adsorption and desorption isotherm of Co-BAND at 298 K.

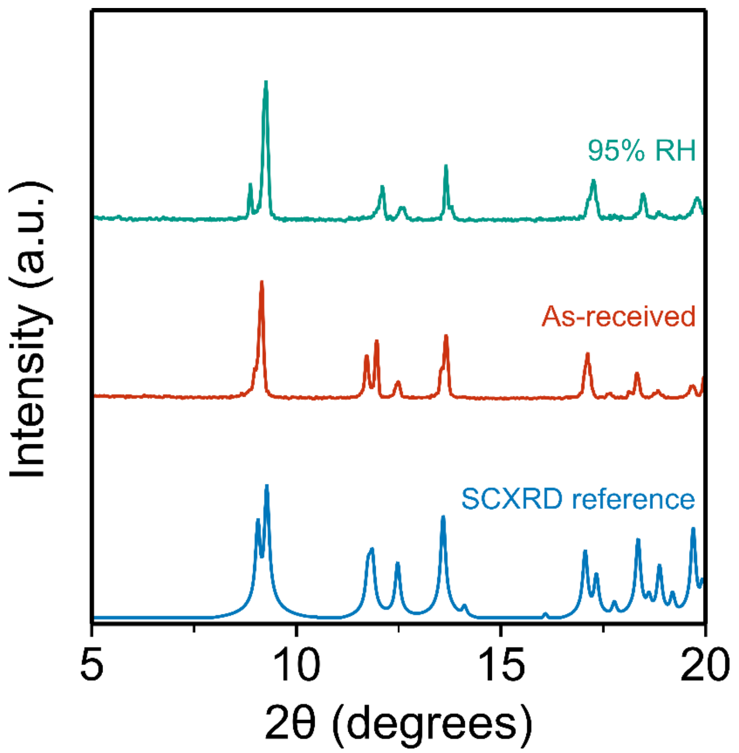

Figure S6. Ex-situ PXRD patterns of Co-BAND after exposure to the noted conditions.

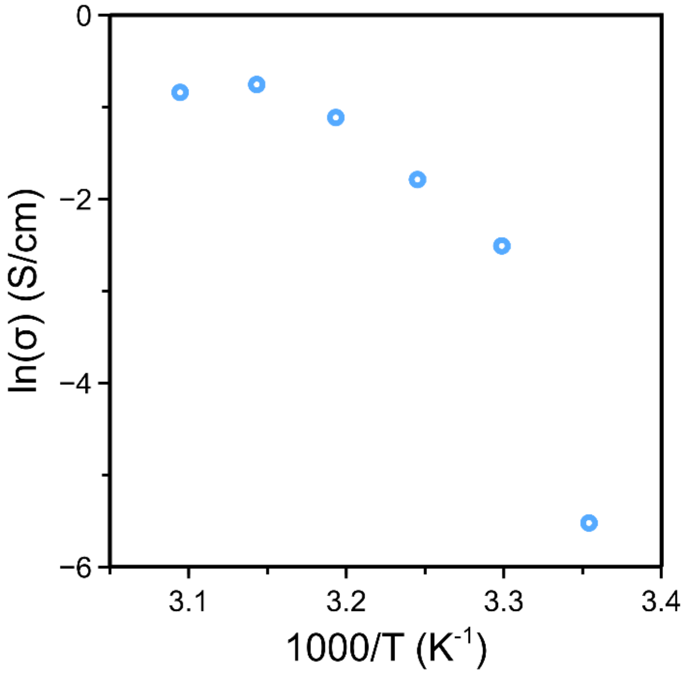

Figure S7. Arrhenius plot of the electrical conductivity of Co-BAND. All data was measured using steady-state, four-point-probe measurements to extract electron-only contributions

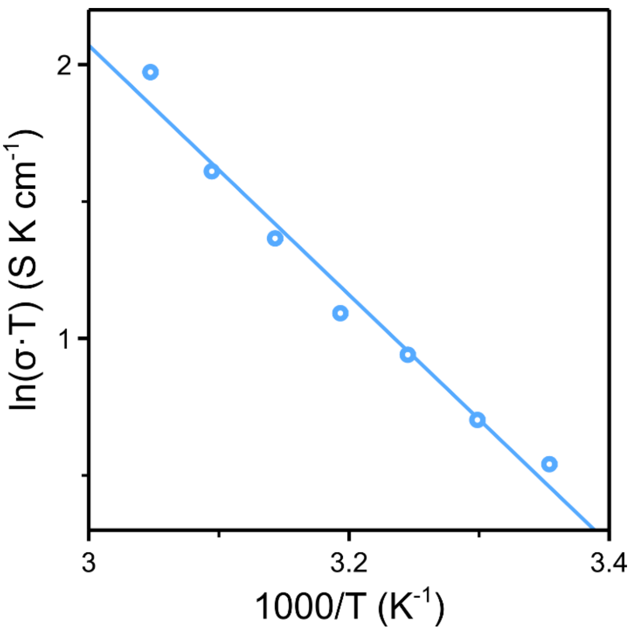

Figure S8. Arrhenius plot of the proton conductivity of Co-BAND.

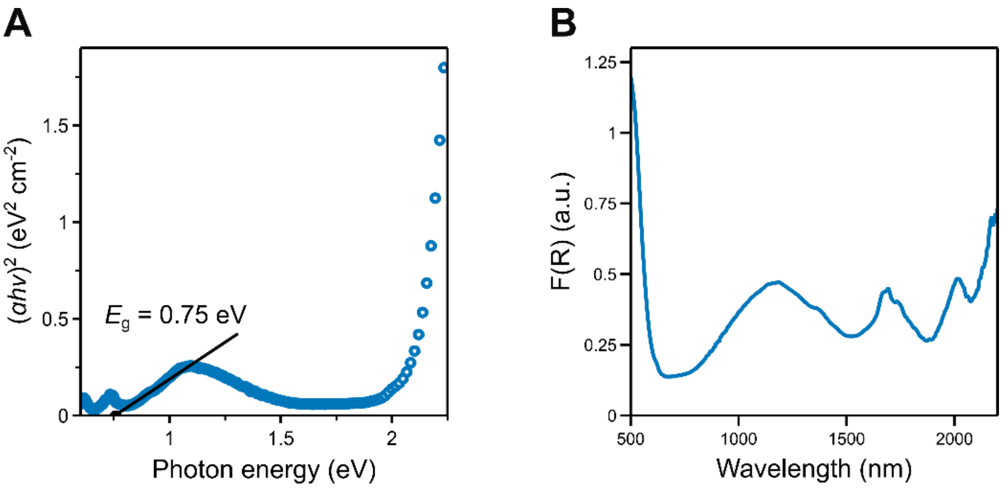

Figure S9. UV-Vis-NIR diffuse reflectance spectrum of Co-BAND. (A) Tauc plot and the estimation of band gap and **(B)** diffuse reflectance spectrum

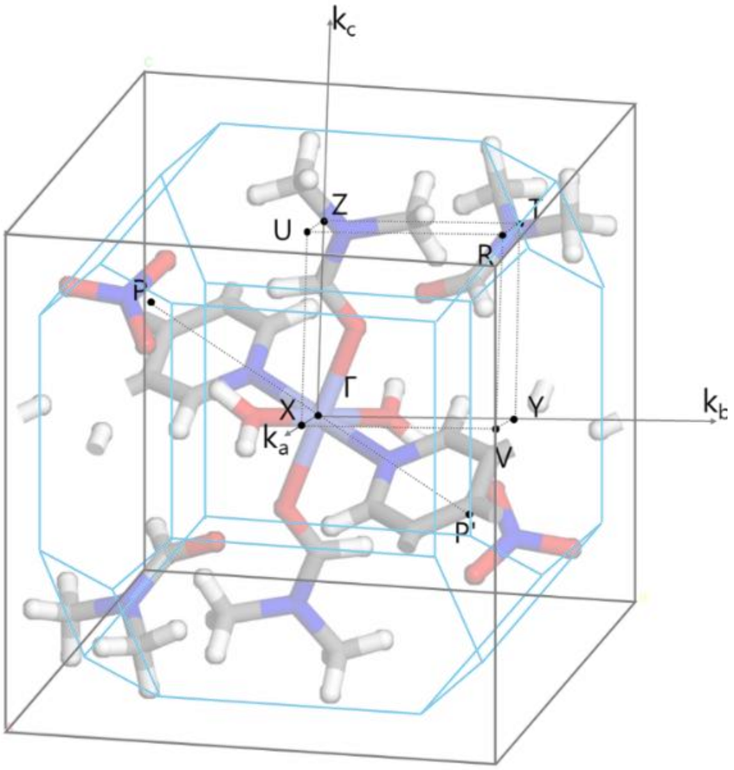

Figure S10. Reciprocal space of Co-BAND used for the density functional theory band structure calculations

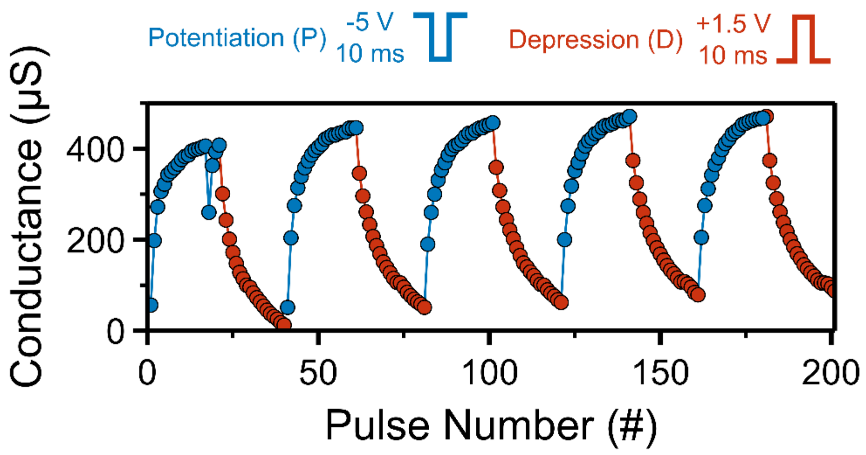

Figure S11. Potentiation and depression of Co-BAND without interpulse delays. The specific pulse conditions are denoted above.

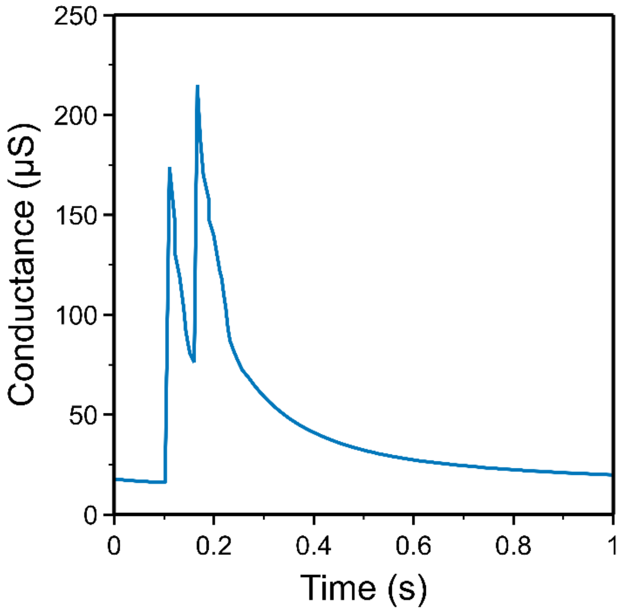

Figure S12. Paired pulse facilitation of Co-BAND using paired stimuli (amplitude: -5 V, pulse width: 10 ms, interpulse interval: 100 ms).

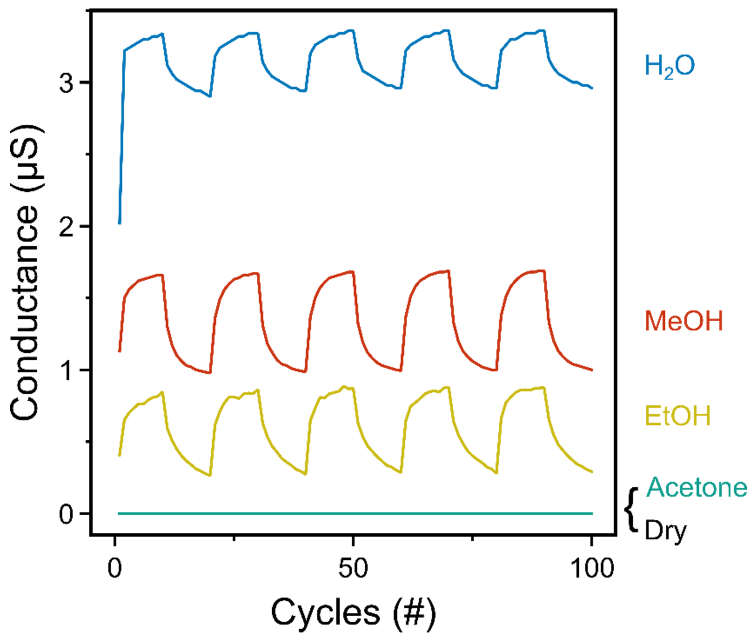

Figure S13. LTP/LTD responses upon different pulse conditions, optimized for EtOH vapor neuromodulator. Set (pulse): -2 V, 50 ms; Reset: 0.5 V, 30 ms; Delay: 100 ms.

Table S1.

Hydrogen bond contacts in Co-BAND based on Steiner's criteria (bond length <3 Å and angle >110°).

Atom1	Atom2	Length (Å)
H8B	O4	2.923
H6OB	O2	1.828
H7B	O3	2.681
H8C	O3	2.894
H7C	O2	2.781
H6OA	O5	2.093
O6	H10C	2.744
O3	H10B	2.88
O3	H11B	2.963
O4	H11B	2.684
O3	H11A	2.685
O4	H11A	2.685

Table S2.
On-off ratios of various humidity-sensitive materials as a function of relative humidity. Reference numbers correspond to those in the main text.

Material	On/Off Ratio	Remarks	Ref.
**Co-BAND**	**1.15×10^6**		This work
Porous BNNFs	1.10×10^7	Nanofiber	28
H_2SO_4@$(NH_2)_2$-MIL-125	10^7	Post-synthetic modification	32
Cs_2SnCl_6/GO	6.5×10^6	Composite	31
$Cs_3Sb_2Br_9$ perovskite	10^5		29
Borophene	1.83×10^3		30

Table S3.

Dissociation energy calculations in eV.

	**Coordinated H_2O in Co-BAND**	**Pristine H_2O**
Bare	-495.91867	-14.22145
Hydrogen	-0.01449	-0.01449
Dissociated form	-491.51946	-7.09679
**Dissociative Energy**	**4.38472**	**7.11017**
